# Modulation of (Homo)Glutathione Metabolism and H_2_O_2_ Accumulation during Soybean Cyst Nematode Infections in Susceptible and Resistant Soybean Cultivars

**DOI:** 10.3390/ijms21020388

**Published:** 2020-01-08

**Authors:** Xi Chen, Shuang Li, Xuebing Zhao, Xiaofeng Zhu, Yuanyuan Wang, Yuanhu Xuan, Xiaoyu Liu, Haiyan Fan, Lijie Chen, Yuxi Duan

**Affiliations:** 1 Nematology Institute of Northern China, Shenyang Agricultural University, Shenyang 110000, China; xchen079@163.com (X.C.); 2019220453@stu.syau.edu.cn (X.Z.); syxf2000@163.com (X.Z.); wyuanyuan1225@163.com (Y.W.); xuanyuanhu007@hotmail.com (Y.X.); liuxiaoyu7805@163.com (X.L.); fanhaiyan6860@163.com (H.F.); chenlijie0210@163.com (L.C.); 2 College of Plant Protection, Shenyang Agricultural University, Shenyang 110000, China; 3 Shaanxi key Laboratory of Chinese Jujube, Yan’an University, Yan’an 716000, China; shuangli@yau.edu.cn; 4 College of Life Sciences, Yan’an University, Yan’an 716000, China; 5 College of Biological Science and Technology, Shenyang Agricultural University, Shenyang 110000, China; 6 College of Sciences, Shenyang Agricultural University, Shenyang 110000, China

**Keywords:** soybean, glutathione, reactive oxygen species, soybean cyst nematode, resistance

## Abstract

In plant immune responses, reactive oxygen species (ROS) act as signaling molecules that activate defense pathways against pathogens, especially following resistance (R) gene-mediated pathogen recognition. Glutathione (GSH), an antioxidant and redox regulator, participates in the removal of hydrogen peroxide (H_2_O_2_). However, the mechanism of GSH-mediated H_2_O_2_ generation in soybeans (*Glycine max* (L.) Merr.) that are resistant to the soybean cyst nematode (SCN; *Heterodera glycines* Ichinohe) remains unclear. To elucidate this underlying relationship, the feeding of race 3 of *H. glycines* with resistant cultivars, Peking and PI88788, was compared with that on a susceptible soybean cultivar, Williams 82. After 5, 10, and 15 days of SCN infection, we quantified γ-glutamylcysteine (γ-EC) and (homo)glutathione ((h)GSH), and a gene expression analysis showed that GSH metabolism in resistant cultivars differed from that in susceptible soybean roots. ROS accumulation was examined both in resistant and susceptible roots upon SCN infection. The time of intense ROS generation was related to the differences of resistance mechanisms in Peking and PI88788. ROS accumulation that was caused by the (h)GSH depletion-arrested nematode development in susceptible Williams 82. These results suggest that (h)GSH metabolism in resistant soybeans plays a key role in the regulation of ROS-generated signals, leading to resistance against nematodes.

## 1. Introduction

The soybean (*Glycine max* (L.) Merr.) has been regarded as the “golden bean” or “miracle bean” [1], and is an important crop that provides a sustainable source of protein and oil worldwide. It is nutritionally rich and has a multitude of industrial uses. The soybean cyst nematode (SCN) (*Heterodera glycines* Ichinohe) is the most economically significant soybean pest that causes substantial damage to soybean production worldwide [2]. Plant parasitic nematodes cause agricultural problems worldwide, resulting in damage estimated at 157 billion US dollars annually [3]. The most effective and environmentally friendly strategy to reduce or eliminate damage from this pest is to breed SCN-resistant soybean varieties [4]. The Peking and PI88788 soybean cultivars are major sources of SCN resistance among the commercial soybean varieties [5,6]. There are two main types of interactions between *Glycine max* and *H. glycines*: The incompatible interaction between resistant soybeans and SCN, which is a resistant reaction, and the compatible interaction between susceptible soybeans and SCN, which is the susceptible reaction. This clustering is based on these cultivars’ vastly different histological responses to *H. glycines* infection [7]. The resistant reaction is divided into two types, that of the PI88788 and Peking cultivars. When *H. glycines* invade roots, the Peking-type resistance reaction is rapid and potent. Juvenile SCN nematode development generally stops at the J2 (juveniles, second stage) stage [8,9,10,11]; however, the resistant reaction of the PI88788-type affects later stages of juvenile nematode development, the J3 (juveniles, third stage) and J4 (juveniles, fourth stage) stages [2]. Klink et al. [12] showed that the expression of genes between *Glycine max* is different between incompatible and compatible populations, affecting a constituent of glutathione synthesis even before it enters the roots. Glutathione synthesis is primarily regulated by the availability of cysteine and, under some conditions, by glycine [13,14,15]. These molecular differences may provide cues regarding what the plant is responding to as the defense response is being engaged. It is clear that the changes in related substances in the initiation of processes of different resistance reactions are important to the elucidation of the mechanism of soybean resistance and the promotion of the breeding of resistant soybean cultivars.

The rapid generation of reactive oxygen species (ROS), such as hydrogen peroxide (H_2_O_2_) and superoxide (O_2_^−^), is the first plant reaction in response to pathogen infection [16]. The effects of H_2_O_2_ on plant development, stress responses, and programmed cell death have been thoroughly investigated [17,18,19]. During the response of tomato plants to fungal infection, a higher generation of ROS, especially H_2_O_2_, appears to be an important element of the disease-resistance mechanism [20,21]. Besides bacterial pathogens, nematodes also induce ROS accumulation in tomato roots [16,22,23]. Silencing *whitefly induced 1* (*Wfi1*), which encodes a respiratory burst oxidase homolog, induces ROS round root-knot nematode (RKN)-infected cells, resulting in the failure of RKN development [24]. In incompatible interactions between pathogens and resistant plants, the oxidative burst is characterized by a rapid and transient accumulation of ROS [25,26]. The hypersensitivity reaction (HR), a typical programmed cell death, is induced to block the spread of pathogens in the host plants after avirulent pathogen invasion [27]. This is associated with the modulation of the enzymatic activities that are involved in the neutralization of ROS production in interactions between resistant tomatoes and root-knot nematode (RKN) [28,29]. The HR phenotypic expression of plant resistance is a phenotypic expression of plant resistance. Most nematode resistance genes in plant species are associated with a typical HR involving the invading nematodes of root cells and the timing of H_2_O_2_ generation, which is one of the most important determinants in blocking successful nematode development [16].

Glutathione (GSH; γ-glutamyl-cysteinyl-glycine) is the major low-molecular-weight thiol in a wide range of organisms. Its synthesis is mainly composed of three key enzymes: γ-glutamylcysteine synthetase (γ-ECS; E.C. 6.3.2.2), glutathione synthetase (GSHS; E.C. 6.3.2.3), and homoglutathione synthetase (hGSHS; E.C. 6.3.2.23). GSH is synthesized from the sequential addition of L-glutamic acid, L-cysteine, and glycine via two ATP-dependent steps catalyzed by γ-ECS and GSHS [30], whereas the synthesis of homoglutathione followed by homoglutathione synthetase catalyzes the addition of β-alanine to γ-GC. GSH is a major cellular antioxidant that maintains intracellular redox homeostasis and may indirectly influence the regulation of cellular processes [30,31,32]. This tripeptide thiol, a marker of oxidative stress, is a major reservoir of reduced sulfur and plays crucial roles in biotic and abiotic stress responses and tolerance in plants [33,34,35]. Its contributions to the tolerance to heavy metals, pathogens, nematode infection, salinity, and drought have been well documented [36,37,38,39]. Under such conditions, plants generally produce GSH, which acts as an antioxidant and redox regulator by combating ROS [40]. It is a considerably established fact that plants have evolved a series of antioxidant molecules and enzymes to protect their cells from oxidative damage [41]. Alscher [42] showed that changes in GSH levels in plants are one of the intrinsic responses of plants to environmental stress.

In the model legume *Medicago truncatula*, (homo)glutathione deficiency alters the nitrogen fixing symbiotic interaction, resulting in a reduction of the formation of root nodules [43]. Pad2.1 is a GSH-deficient mutant of *Arabidopsis thaliana* discovered by Parisy et al. It has been shown that adequate levels of GSH in *A. thaliana* are crucial for the limitation of virulent *Pseudomonas syringae* development and the establishment of disease resistance to many pathogens [44,45]. The high level of (h)GSH in root nodules suggests that (h)GSH is involved in the protection of the nitrogen-fixing nodules against the oxidative stress of the active nodule metabolism [46,47]. The transcriptomic response of (h)GSH-deficient plants to *Sinorhizobium meliloti* infection has demonstrated a downregulation of genes that are involved in meristem formation and an increased expression of several genes involved in the early plant defense reaction [48]. A deficiency in (h)GSH impairs nematode reproduction and development during the *Medicago truncatula*–*Meloidogyne incognita* interaction, and the tripeptide thiol may play a key role in the regulation of metabolic activity in giant cells [39].

In this work, we designed time-course experiments to analyze the mechanism of (h)GSH and H_2_O_2_ production during the plant–nematode interaction to compare plant responses of susceptible and resistant soybeans upon exposure to *H. glycines*. We showed that there were low levels of (h)GSH metabolism in resistant cultivars relative to those in susceptible cultivars. Resistant plants induced intense ROS generation to arrest SCN advancement. We further demonstrated that (h)GSH deficiency led to a strongly delay in nematode development and oxidative burst. (h)GSH is considered a major antioxidant and is inter-linked with oxidants, such as (O_2_^−^) and H_2_O_2_, which are also present inside cells, especially under stress conditions [49,50]. Plants are equipped with various antioxidant defenses that help to modify H_2_O_2_ production and activate host resistant defense. Therefore, the timing of the (h)GSH–H_2_O_2_ interaction in different resistant cultivars is a consequence of resistance or a hallmark of blocking successful nematode development.

## 2. Results

### 2.1. Root Penetration

**Figure 1:**
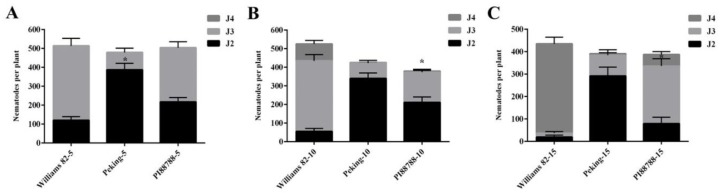
Analysis of nematode developmental stages in roots of Peking and PI88788 (resistant) and Williams 82 (susceptible) assayed at 5, 10, and 15 dpi (days post-inoculation). Roots were dissected 5 (**A**), 10 (**B**), and 15 (**C**) dpi, and nematode developmental stages (juveniles, second stage (J2), J3, and J4) were analyzed. The number of J4 include cysts. Data (nematodes from 15 plants produced in three different biological experiments) are represented by the mean ± standard error. * indicates a statistically significant difference (*p* < 0.05).

To assess the time-course of GSH involvement during the plant–nematode interaction, we analyzed the development of nematodes in susceptible and resistant soybean lines. The second-stage infective juveniles were able to penetrate the roots of both the resistant cultivars, Peking and PI88788, as well as the susceptible Williams 82 cultivar that was inoculated with SCN as a control. The results showed considerable numbers of J2 at different stages of infection. At 5 dpi (days post-inoculation), the number of J2 in Peking was greater than that in the control for each individual root, and most of the juveniles in the roots were still at the J2 stage (Figure 1A). The two resistant lines exhibited J2 development arrested at 5 dpi. However, the resistant reaction of PI88788 affected a number of juvenile nematodes at the J3 and J4 stages between 10 and 15 dpi (Figure 1B,C). The numbers of J3 among the PI88788 roots were significantly greater than those in the Peking roots; however, the number of J4 individuals was significantly lower than that of the control at 15 dpi (Figure 1B,C).

### 2.2. (h) GSH Metabolism Was Modified in Nematode-Induced Soybean Roots

**Figure 2:**
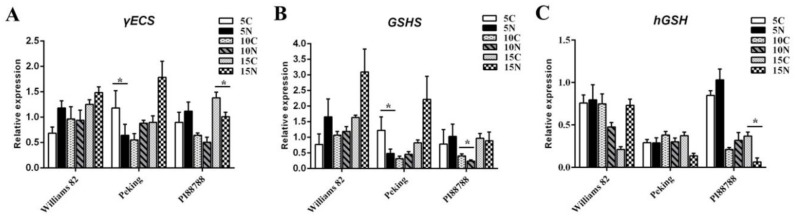
qRT-PCR expression analysis of the genes involved in the glutathione and homoglutathione synthesis pathways in Peking and PI88788 (resistant) and Williams 82 (susceptible) soybean roots. The expression levels of γ-glutamylcysteine synthetase (γECS) (**A**), glutathione synthetase (GSHS) (**B**), and homoglutathione synthetase (hGSHS) (**C**) were analyzed in uninfected and infected roots at 5, 10 and 15 days post infection. Graphs show gene expression in infected roots as a multiple of that in the uninfected roots. C and N represent uninfected and infected roots, respectively. Data (technical triplicates of three biological experiments) are reported as means ± standard error. * indicates a statistically significant difference (*p* < 0.05).

To determine the potential involvement of GSH in soybeans that are resistant against SCN, (h)GSH metabolism was differentially analyzed between susceptible and resistant roots at different time points. First, the expression level of both the *γECS* and *GSHS* genes in Peking were significantly lower than that in uninfected roots at 5 dpi (Figure 2A,B). However, a significant difference between the expression of *γ-ECS* and *GSHS* was observed between 10 and 15 dpi, in which they were up-regulated after SCN infection (Figure 2B,C). No significant difference in the expression of *hGSH* was observed between infected and uninfected roots (except at 15 dpi). However, the expression levels of both the *γECS* and *hGSH* genes in PI88788 were markedly down-regulated at 15 dpi (Figure 2A,C), and the expression of the *GSHS* gene was significantly lower than that of the controls at 10 dpi (Figure 2B). In contrast, the expression of the *γECS* and *GSHS* gene expression levels in Williams 82 was always induced during SCN infection at each time point according to qRT-PCR relative to that of the control. Interestingly, at 15 dpi, the expression of the *hGSH* gene was lower after SCN infection than that in the uninfected Williams 82 roots (Figure 2C).

**Figure 3:**
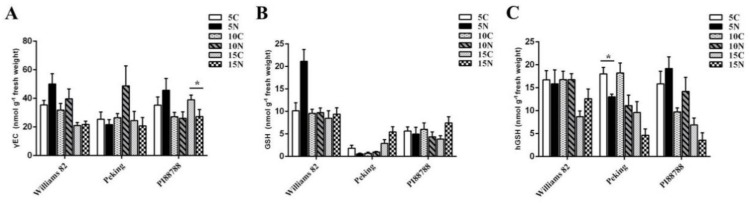
Time-course quantification of γEC and (h)GSH in Peking and PI88788 (resistant) and Williams 82 (susceptible) soybean roots. Infected and uninfected root γ-glutamylcysteine (γEC) (**A**), glutathione (GSH) (**B**), and homoglutathione (hGSH) content (**C**) at 5, 10, and 15 days post infection. C and N represent uninfected and infected roots, respectively. The data (six samples from three independent biological experiments) are reported as the mean ± standard error. * indicates a statistically significant difference (*p* < 0.05).

We tested whether the changes in the expression of the genes involved in (h)GSH synthesis were correlated with the levels of γ-EC, GSH, and hGSH. In Williams 82, Peking, and PI88788, an HPLC analysis showed that quantitative trends were roughly similar to that of the gene expression of *γ-ECS* and *(h)GSHS*. However, differences in metabolite contents were less marked than the differences between gene expression levels. There was no significant difference in the γEC and GSH contents of resistant soybeans between infected and non-infected roots at 5 and 10 dpi (Figure 3A,B). The level of hGSH declined in resistant Peking roots during the three time points. At 15 dpi, hGSH was lower than that in the uninfected roots of resistant PI88788 (Figure 3C). The GSH level was lower in resistant roots than that in susceptible roots. Furthermore, the level of GSH in resistant Peking roots with SCN was 71% lower than that in uninfected roots at 5 dpi. At 10 dpi, there was a 28% reduction in the GSH level of resistant PI88788 roots relative to that in the uninfected control roots (Figure 3B).

### 2.3. Quantitative Detection of ROS Production

**Figure 4:**
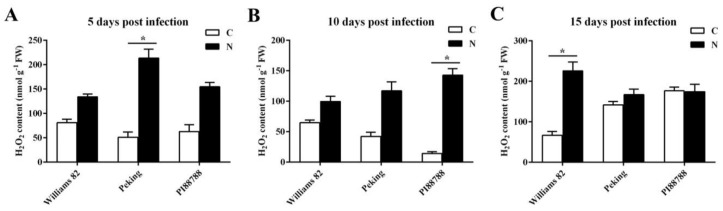
Accumulation of reactive oxygen species (ROS) after soybean cyst nematode (SCN) infection in the roots of Peking and PI88788 (resistant) and Williams 82 (susceptible) soybeans assayed at 5, 10, and 15 days post infection. H_2_O_2_ accumulation was determined at 5 (**A**), 10 (**B**), and 15 (**C**) days after SCN infection. C and N represent uninfected and infected roots, respectively. FW: fresh weight. Values are mean ± standard error of combined data from two separate three-fold replicated experiments. * indicates statistically significant differences between uninfected and infected roots (*p* < 0.05).

Since GSH plays a critical role in the control of the cellular redox status in plant tissues, we examined H_2_O_2_ levels in susceptible and resistant lines. We found that H_2_O_2_ accumulation was induced by SCN in the roots of susceptible and resistant soybeans. Importantly, the most significant increase in levels of H_2_O_2_ accumulation was recorded in the resistant Peking roots that were infected with SCN at 5 dpi (Figure 4A). A high increase in H_2_O_2_ accumulation at 10 dpi was observed in the root tissue of resistant PI88788 roots, as shown in Figure 4A. Interestingly, at 15 dpi, the oxidative burst was detected in the roots of susceptible Williams 82, whereas no significant difference in resistant Peking and PI88788 roots was observed in response to SCN infection (Figure 4C).

### 2.4. (h)GSH Deficiency Impairs Nematode Development and Reproduction

**Figure 5:**
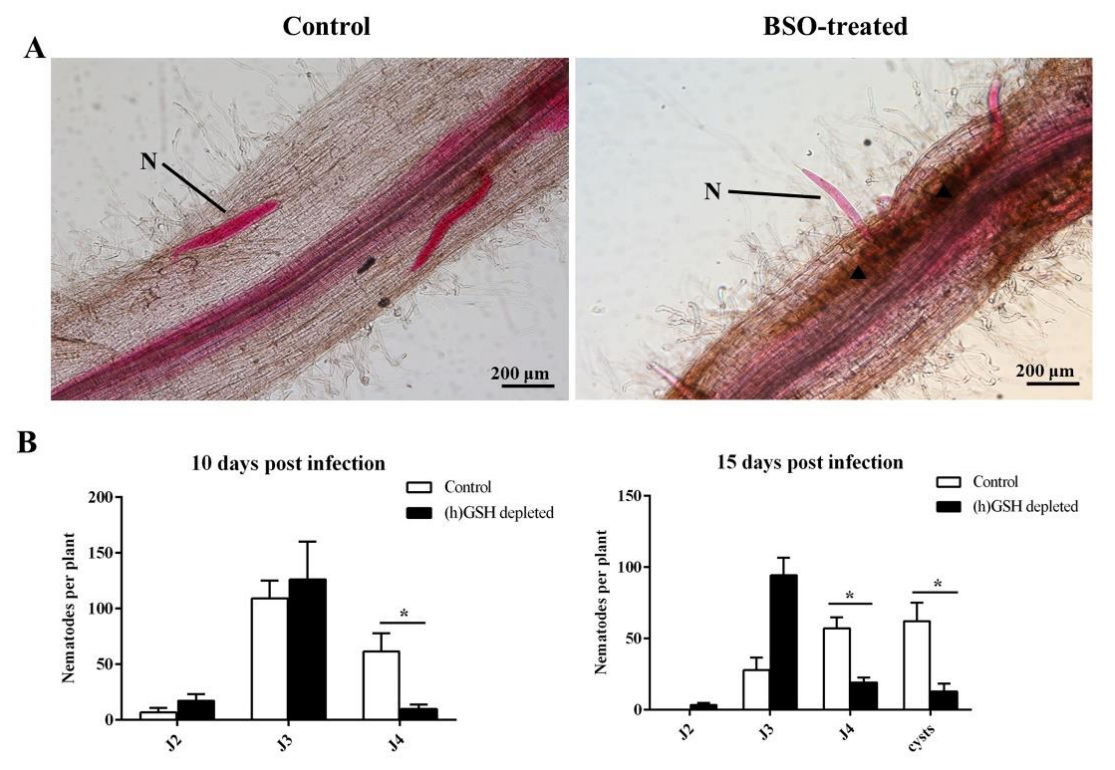
Analysis of nematode developmental stages in (h)GSH-depleted roots. (**A**) Penetration and development of *H. glycines* in the control and (h)GSH-depleted roots by acid fuchsin-stained at 5 days post infection. (**B**) Effects of L-buthionine-[S–R]-sulfoximine (BSO) treatment on nematode developmental stage (juveniles and cysts) were analyzed at 10 and 15 days post infection. Data (nematodes from 15 plants produced in three different biological experiments) are represented by mean ± standard error. * indicates a statistically significant difference (*p* < 0.05). N, nematode; ▲, hypersensitivity reaction (HR)-like cell death.

During plant-nematode interactions, the depletion of (h)GSH content impaired root-knot nematode growth and reproduction [39]. Treatment with L-buthionine-[S–R]-sulfoximine (BSO), a specific inhibitor of (h)GSH synthesis, on nematodes (1 mM) and plants (0.1 mM) was used to reduce the total (h)GSH in roots, as previously described [43], in order to eliminate the hypothesis that BSO might be involved in the direct impairment of nematodes. To test the effect of (h)GSH deficiency on SCN attraction, invasion, and development after inoculation, acid fuchsin-stained nematodes were observed at 5 dpi. We found mostly J2 individuals touching the roots of (h)GSH-depleted plants, whereas J3 individuals occurred in the control roots (Figure 5A). BSO treatment led to a 66% reduction of J3 production relative to the control plants at 5 dpi (Figure S1). Importantly, the susceptible line, Williams 82, exhibited HR-like cell death around the area of nematode infection after BSO treatment. HR is programmed cell death and is a common plant reaction against different pathogens in resistant cultivars. Compared to the roots of each control and (h)GSH-depleted plants upon *H. glycines* infection, the number of nematodes at later developmental stages (second, third, and fourth stages, as well as cysts) was counted (Figure 5B). At 10 dpi, the number of J4 was much lower in the (h)GSH-depleted roots than that in the control. The numbers of nematodes in (h)GSH-depleted roots at 15 dpi showed that an average of 94 J3 individuals was examined per root, whereas only 28 nematodes were observed in each control root. Significantly fewer J4 juveniles were observed in the BSO-treated roots than that in those of the control. We also found that the number of cysts was significantly reduced by 80% after BSO treatment when compared to that of the control. These results suggest that nematode development is affected by (h)GSH depletion.

### 2.5. Association between (h)GSH Deficiency and H_2_O_2_ Accumulation in Response to SCN

**Figure 6:**
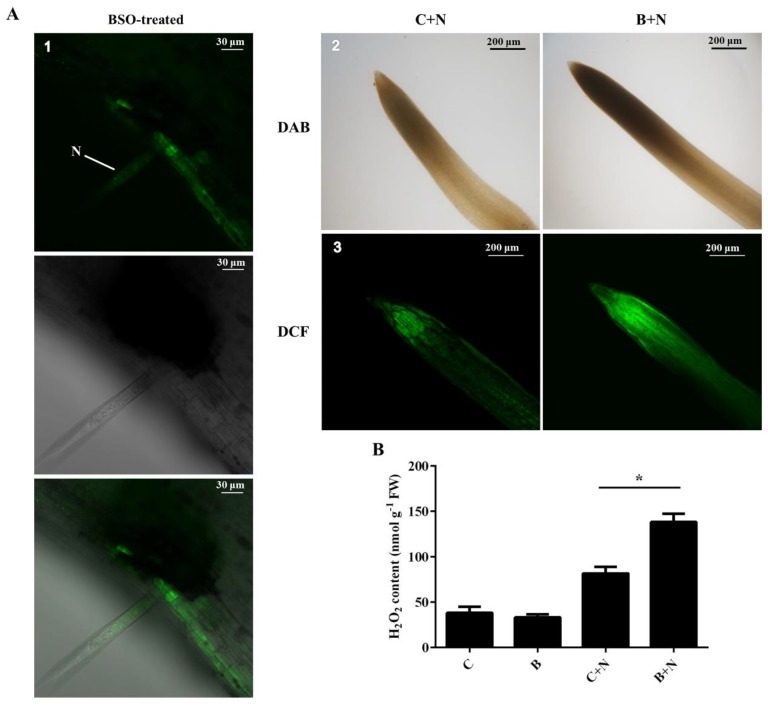
Analysis of reactive oxygen species accumulation in (h)GSH-depleted roots. (**A**) Histochemical analysis of the production of H_2_O_2_ using 2,7-dichlorofluorescein diacetate (DCF) (A1 and A3) and 3,3′-diaminobenzidine (DAB) (A2) in (h)GSH-depleted roots of Williams 82 (susceptible) at 5 d post-inoculation. (**B**) The levels of H_2_O_2_ were determined after 5 d in uninfected roots (**C**), BSO-treated uninfected roots (C and N), BSO-treated uninfected roots (**B**), and BSO-treated infected roots (B and N). FW, fresh weight. Data (technical triplicates of three biological experiments) are given as mean ± standard error. * indicates a statistically significant difference (*p* < 0.05). N, nematode.

To determine whether GSH is linked to a delay or an arrest in nematode development by regulating H_2_O_2_ production, we found that the 2,7-dichlorofluorescein diacetate (DCF) signal intensity highly increased at the site of nematode infection in (h)GSH-depleted plants by using DCF as a probe for H_2_O_2_ (Figure 6A1). We visualized ROS with 3,3′-diaminobenzidine (DAB) in order to compare the accumulation of H_2_O_2_ in SCN-infected lines under BSO treatment. (h)GSH deficiency affected DAB staining in invaded roots (Figure 6A2). To confirm the specificity of DAB-visualized ROS production, we found that 2,7-dichlorofluorescein diacetate (CM-H_2_DCFDA) fluorescence was more intense in the (h)GSH-depleted roots treated with SCN than those in the control roots that were treated with DCF staining (Figure 6A3). A quantitative analysis showed that the production H_2_O_2_ was similar between the control and (h)GSH-depleted roots in the absence of SCN (Figure 6B). Strikingly, the production of H_2_O_2_ was significantly induced by SCN at 5 dpi in the (h)GSH-depleted roots, as evidenced by histochemical staining with DAB and DCF (Figure 6A). Thus, these results indicate that GSH may be involved in the regulation of H_2_O_2_, which is induced in response to SCN infection.

## 3. Discussion

(h)GSH plays a major role in antioxidant defense, the detoxification of xenobiotics, and tolerance to abiotic and biotic stresses [51]. A threshold (h)GSH concentration is essential for plant and organ development [52,53]. Transcriptomic analysis has shown the regulation of (h)GSH metabolism both in plant development and defense responses [48]. Surprisingly, in the current study, post-infection resistance was associated with (h)GSH metabolism in the two types of mechanisms for SCN resistance in soybeans. The downregulation of *γECS* transcript levels was observed in resistant roots, whereas this gene should regulate the level of *(h)GSHS*. Decreased *GSHS* transcription might affect the growth of J2 in resistant Peking roots. The hGSH content was significantly lower in the infected roots than in the PI88788 roots, which impaired nematodes at later developmental stages. In addition, the accumulation of GSH in the susceptible Williams 82 roots might have been caused by the nematodes, as GSHS has been identified as a protein that is secreted by *Meloidogyne incognita* [54]. The difference between susceptible and resistant varieties may be the effects on the resources that are required for the completion of the nematode life cycle, including nematodes secreting multiple redox- and (h)GSH-regulated proteins [54,55].

The formation of ROS is a common feature of abiotic and biotic stress reactions. It plays an important role in plant defense responses, and the activity of ROS detoxifying enzymes is often suppressed in resistant plants during pathogen attacks [56]. During the incompatible interaction between *Arabidopsis thaliana* and *H. glycines*, symptoms of HR and the production of H_2_O_2_ have been documented [57]. A number of studies have shown that the intensity and time of ROS generation may trigger opposite effects in plants [21,58]. Interestingly, in the comparison between infected and uninfected resistant soybean roots, we observed that the magnitude of H_2_O_2_ production was significantly increased in different periods of nematode development. During the later stages of infection, the production of H_2_O_2_ was highly induced by SCN in susceptible Williams 82, but no significant difference was observed with that in resistant roots. It is possible that in susceptible roots, the females had reached reproductive maturity and started to lay eggs, inducing H_2_O_2_ accumulation, whereas nematodes failed to produce any egg masses in resistant roots. Indeed, the *de novo* biosynthesis of GSH is one of the important defense responses during ozone-exposed processes [59]. The control of plant cell redox status through the modification of (h)GSH content may be a key regulator of nematode development. It is clear that GSH metabolism is involved in the oxidative events and affected the activity of the main enzymes that are responsible for ROS detoxification [60]. We showed that root (h)GSH deficiency strongly impaired nematode development. Additionally, HR-like cell death and H_2_O_2_ production around the area of nematode infection were observed in (h)GSH-depleted roots. However, death of syncytia frequently occurs in resistant soybeans upon SCN infection [61].

Thus, the level of GSH in cultivars that are resistant to *H. glycines* leads to a decrease in the degree of the cellular GSH pool and, thus, a change in the cellular redox system in the plant, which is indicated by an increase in ROS formation. It is possible that the dramatic increase in the concentration of ROS that was detected in the incompatible relationship could be related to ROS-mediated localized cell death in root tissues around the nematodes, which prevents the formation of a developed feeding site, thus leading to resistance. For example, H_2_O_2_ can trigger HR in incompatible interactions [62]. Different subcellular antioxidants between the two types of mechanisms for SCN resistance in soybeans might contribute to local redox changes. Additionally, the suppression of ROS detoxifying mechanisms is also essential for the onset of cell death [19]. Feeding cells are also disassociated from surrounding tissue in resistant plants after infection with RKNs [63,64]. As a result, resistant plants produce more ROS, and the accumulation of these components may lead to HR.

In conclusion, we report that (h)GSH metabolism differs between susceptible and resistant soybean roots upon SCN infection, with a decrease in resistant soybeans. This difference may be explained by the observation of lowered GSH synthesis capacity and increased redox states. There was a significant cellular redox status difference between (h)GSH-depleted roots and that of controls, strongly suggesting that H_2_O_2_ production was affected by (h)GSH depletion. It is known that a higher generation of H_2_O_2_ in tomato plants, as a result of fungal infection, appears to be an important factor of disease-resistance mechanisms [20,21]. This pattern of H_2_O_2_ production correlates well with the process of nematode development that was found in the resistant and susceptible soybean roots. Additionally, the BSO-mediated depletion of GSH-induced ROS accumulation around SCN-infected cells, resulting in the failure of SCN development in the roots. Different subcellular antioxidants in plants may be linked to the modification of ROS production and associated metabolism, or by these antioxidants may be otherwise linked by driving changes in the redox state of components to impair the growth of nutrients and the development of nematodes in the host plant [65]. An analysis of *Caenorhabditis elegans* by using γECS-RNAi or gene deletion showed that GSH depletion is not lethal to larvae or embryonic worms and does not induce slow growth or female sterility [66,67,68]. The low level of (h)GSH in roots may cause the accumulation of intracellular ROS, resulting in an unsuitable redox state that inhibits nematode development. This mechanism appears to be novel for SCN resistance. The management of the plant cell redox system via the modification of (h)GSH content may be a key regulator during the interaction between resistant soybeans and soybean cyst nematodes. Therefore, the reduction of (h)GSH availability in infected roots is a potentially useful strategy for pest management. Taken together, it can be stated that (h)GSH metabolism differences and H_2_O_2_ generation between resistant cultivars might lead to HR responses in the nematode–host interaction. However, no single mechanism has been unequivocally proven to operate in plant cells. The redox state and GSH affect the function of many enzymes through the level of posttranslational modification control [69].

In this respect, it is worth evaluating the expression levels of genes that code for enzymes that are involved in the formation of ROS and ROS scavenging. The GSH/GSSG ratio decreased under high temperature stress, which may be due to the oxidation of GSH to GSSG in the process of scavenging ROS [70]. In this way, we could obtain a relatively comprehensive view of the intricacies of redox changes that occur upon SCN infection in resistant soybeans. Additionally, it is well known that salicylic acid (SA) enhances the HR that protects the plant against the pathogen [71]. Vital to signal transduction processes that are associated with defense responses appears to be the interaction between glutathione, H_2_O_2_, and SA [72,73]. Further research should determine how GSH takes an active part in the cross-communication with other signaling pathways, such as SA, jasmonic acid (JA), and ethylene (ET), in induced defense responses, as well as how GSH modulates the expression of defense-related genes in plant–nematode interactions, as evidenced earlier in other plant–pathogen interactions [74].

## 4. Materials and Methods

### 4.1. Plant Material, Growth Condition, and Treatments

Seeds of *G. max* (two SCN-resistant cultivars, Peking and PI88788, and an SCN-susceptible control line, Williams 82) were used for all experiments. All seeds (stored at the Nematology Institute of Northern, Shenyang, China) were surface-sterilized with 0.5% (*w/v*) NaClO for 5 min and washed several times with sterile distilled water (SDW) to remove residual NaClO [75]. For the 0.1 mM L-buthionine sulfoximine (BSO)-treated experiment, the seeds of susceptible Williams 82 were germinated in the presence or absence of BSO. Then, the sterilized seeds were planted in pots containing soil and sand at a 1:1 ratio (*v/v*). Seeds were grown with a day/night temperature of 23 °C/20 °C and a photoperiod of 16 h.

### 4.2. Nematode Inoculum

An infested soil and water mixture was stirred. The soil suspension was passed through nested 850-μm-pore over 250-μm-pore sieves with *H. glycines* cysts. Eggs of *H. glycines* were extracted from the cysts by grinding the sediment that was collected on the 250 μm-pore sieves [76]. Eggs were isolated from the soil with a 1.9 M sucrose solution via 1118 g for 60 s. The eggs were treated with 0.5% (*w/v*) NaClO for 5 min for surface disinfestation and washed several times with sterile distilled water (SDW) [75]. Free eggs were incubated in a chamber dip with 3 mM ZnSO4 in the dark until second-stage juveniles (J2) hatched and were collected daily. For the BSO-treated experiment, nematodes were incubated for 4 h in 1% resorcinol and 1 mM BSO with 1% resorcinol as a control. Plants were grown in the presence or absence of BSO (0.1 mM) in a growth chamber until the end of the experiment. For nematode infection, 2000 freshly hatched J2 individuals mixed with 0.2% agar were added to each soybean root. After 5, 10, and 15 days post inoculation (dpi), the roots of infected soybeans were harvested, with uninfected roots under the same conditions serving as controls. All collected roots were immediately frozen in liquid nitrogen. All above experiments were repeated at least three times. These samples were then stored at −80 °C in a deep freezer until the following experiments.

### 4.3. Root Penetration Studies

To observe the development of *H. glycines* in different soybean lines, the fresh roots were stained with acid fuchsin to monitor nematode invasion [77] at 5, 10, and 15 dpi. The roots were carefully removed from the soil, and cysts were counted directly. After staining, the SCN juveniles from the J2–J4 stages inside the roots and cysts were morphologically examined and counted with a microscope (Nikon, Tokyo, Japan). An analysis of variance (ANOVA) was used to compare the penetration rate between resistant and susceptible roots. Five biological replicates were selected for each sampling time point.

### 4.4. Total RNA Extraction and Gene Expression Analysis

The total RNA for qRT-PCR was isolated from the collected roots of each biological replicate by using an RNeasy Kit (Invitrogen) following the manufacturer’s instructions. DNA impurities in the isolated RNA were digested before synthesizing the cDNA by adding gDNA Eraser (TAKARA, Dalian, China) and incubated for 2 min at 42 °C. Then, the first-strand cDNA was synthesized from 1 μg of total RNA with the Prime Script™ RT Reagent Kit (TAKARA, Dalian, China), following the manufacturer’s instructions. Quantitative PCR reactions were performed with a Bio-Rad system (CFX Connect TM Real Time PCR Detection System, CA, USA) and TAKARA SYBR Green Master mix (TAKARA, Dalian, China) in a 10 µL reaction volume. A single 10 μL PCR included 5 μL of TAKARA SYBR Green Master mix (TAKARA, Dalian), 1 μL of cDNA template, and 10 μM each of forward and reverse primers (sequences used are described in Table S1). The actin gene *GmActin11* was used as an internal control [78]. The PCR cycling parameters were as follows: pre-incubation at 95 °C for 2 min, 39 cycles of 95 °C for 5 s, 60 °C for 30 s, and 95 °C for 5 s. PCR reactions for each of three biological replicates were performed in technical triplicate. The relative expression level was normalized with the 2^−ΔΔCt^ method [79].

### 4.5. γ-Glu-Cys and (h)GSH Determination

Commercial GSH (Sigma, St. Louis, America) and γ-glutamyl-cysteine (γ-Glu-Cys) (Aladdin, Shanghai, China) were used as standards. The hGSH that was used as a standard was synthesized by GL Biochem (Shanghai, China). The (h)GSH and γ-Glu-Cys calibration solutions (400 μg/mL) were prepared in water. The tissue samples of roots after freeze-drying were ground in liquid nitrogen with a porcelain mortar. 1 g sample of the tissue was homogenized by ultrasonication in 15 mL, ice-cold ultra-pure water. This homogenate was then centrifuged at 19,760× *g* for 15 min. The solutions were passed through 0.45 μM Millipore filters and loaded into the autosampler tray.

Samples were analyzed with a 1260 series HPLC system (Agilent Technologies, Massy, France), including an autosampler, a binary pump, and an AQ-C18 column (150 × 4.6 × 10 μm, Ultimate). The flow-rate of 1.0 mL/min was achieved with an elution gradient composed of solvent A (0.1% trifluoroacetic acid in water) and solvent B (methanol). The separation was performed under isocratic conditions with a 98% mobile phase A at a column temperature of 35 °C. The total analysis time was 15 min. For comparative studies, solutions of 8, 40.0, 80. 0, 120. 0, and 160. 0 μg/mL of (h)GSH and γ-Glu-Cys were analyzed. The curves were constructed by plotting the sum of peak areas for the HPLC method versus the analyte concentration. Linear regression analysis was used to determine the slope, intercept, and correlation coefficient (*r*^2^, γ-Glu-Cys:0.9264, GSH:0.9991, hGSH:0.9966).

### 4.6. H_2_O_2_ Quantification and Cytochemical Detection

Portions of the uninfected control roots and 5, 10, and 15 dpi roots that were infected with SCN were excised, and 100 mg of tissue for each was extracted with 0.1% trichloroacetic acid (TCA) at 4 °C. The mixture was then centrifuged (17,000× *g* for 15 min), and the supernatant was mixed with 0.5 mL of 100 mM phosphate buffer saline (pH 7.4) and 1 mL of 1 M KI for 1 h at 28 °C in the dark. The H_2_O_2_ content was determined by measuring at 390 nm with a Thermo Multiskan GO Enzyme Marker.

To visualize H_2_O_2_, a type of reactive oxygen species (ROS), roots from susceptible and resistant soybeans were collected at 5, 10, and 15 d after nematode inoculation and stained with a 3,3′-diaminobenzidine (DAB) solution containing 1 mg/mL of DAB (pH 5.0) at room temperature for 4 h. The detached roots were boiled in 90% (*v/v*) ethanol for 10 min, stored in acidified glycerin, and photographed (BX53; Olympus Co., Tokyo, Japan) [80].

To detect ROS production by using a confocal laser-scanning microscope (CLSM) (excitation 488 nm, emission 521 nm), a membrane-permeable probe and CM-H_2_DCFDA were used. Root segments of susceptible cultivars in the presence or absence of BSO, uninfected and infected with nematodes (5, 10, and 15 dpi), respectively, were incubated with CM-H_2_DCFDA (10 µM) in phosphate-buffered saline for 90 min at 4 °C [81]. The samples were then washed several times with KCl (0.1 mM) and CaCl_2_ (0.1 mM) to remove excess CM-H_2_DCFDA. The samples were imaged with an OLYMPUS CLSM FV3000.

### 4.7. Statistical Analysis

All statistical analyses were performed by using an ANOVA with the SPSS statistical software package (Version 22.0; SPSS, Inc., Chicago, IL, USA) and Microsoft Office Excel 2010. The data are provided as means and standard error of three independent biological experiments. The significance of the results was tested by using Student’s *t*-test (*p* < 0.05).

## Supplementary Materials

Supplementary materials can be found at https://www.mdpi.com/1422-0067/21/2/388/s1. Supplementary Table S1 shows the genes and primers used in this study, Supplementary Figure S1 shows the number of nematodes inside the roots of the (h) GSH-depleted and control plants at 5 d post-inoculation (dpi).

## Abbreviations

γ-Glu-Cys	γ-Glutamylcysteine
GSH	Reduced glutathione
GSSG	Oxidized glutathione
hGSH	homoGlutathione
*γ-ECS*	γ-Glutamylcysteine synthetase
*GSHS*	Glutathione synthetase
*hGSHS*	homoGlutathione synthetase
ROS	Reactive oxygen species
BSO	L-buthionine sulfoximine
H_2_O_2_	Hydrogen peroxide
O_2_^−^	Superoxide
HR	Hypersensitive reaction
RKN	Root-knot nematode
SCN	Soybean cyst nematode
J2	Second-stage juvenile
J3	Third-stage juvenile
J4	Fourth-stage juvenile
dpi	Days post-inoculation
NaOCl	Sodium hypochlorite
SDW	Sterile distilled water
HPLC	High-performance liquid chromatography
qRT-PCR	Quantitative reverse transcription-PCR
CLSM	Confocal laser-scanning microscope
SA	Salicylic acid
JA	Jasmonic acid
ET	Ethylene
