# Supplementary material for: Modulation of (Homo)Glutathione Metabolism and H2O2 Accumulation during Soybean Cyst Nematode Infections in Susceptible and Resistant Soybean Cultivars
Source: Int J Mol Sci. 2020 Jan 8;21(2):388. doi: 10.3390/ijms21020388 (PMC7013558; doi:10.3390/ijms21020388)
Supplement: Supplementary file 1 [file ijms-21-00388-s001.pdf]

Table S1. PCR primers used for qRT-PCR assays.

Gene name	Forward primer (5’-3’)	Reverse primer (5’-3’)
γ-ECS	TGAAGACACCATTCCGAGAC	ACACCTGTTCTAACCACCTC
GSHS	CTTATGAAGGTGGAGTTTTGCC	GCCTCTTTTCTTCTTTCACTGG
hGSHS	ACCTTTGACCACCAACTCTG	CACCCTTGCTTCCAATGACT
Actin11	CGGTGGTTCTATCTTGGCATC	CGGTGGTTCTATCTTGGCATC

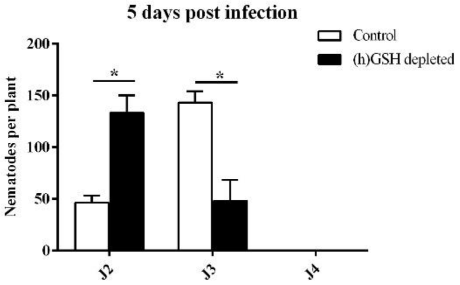

Figure S1. Number of nematodes inside the roots of the (h)GSH-depleted and control plants at 5 d post-inoculation (dpi). Data (nematodes from 15 plants produced in three different biological experiments) are represented by mean ± standard error. * indicates a statistically significant difference ($P < 0.05$).
